# Genome-Wide Association Study of Serum 25-Hydroxyvitamin D in US Women

**DOI:** 10.3389/fgene.2018.00067

**Published:** 2018-03-01

**Authors:** Katie M. O'Brien, Dale P. Sandler, Min Shi, Quaker E. Harmon, Jack A. Taylor, Clarice R. Weinberg

**Affiliations:** ^1^Biostatistics and Computational Biology Branch, National Institute of Environmental Health Sciences, Durham, NC, United States; ^2^Epidemiology Branch, National Institute of Environmental Health Sciences, Durham, NC, United States

**Keywords:** 25-dihydroxy vitamin D, genome-wide association study, single nucleotide polymorphism, vitamin D binding protein, CYP2R1

## Abstract

Genetic factors likely influence individuals' concentrations of 25-hydroxyvitamin D [25(OH)D], a biomarker of vitamin D exposure previously linked to reduced risk of several chronic diseases. We conducted a genome-wide association study of serum 25(OH)D (assessed using liquid chromatography-tandem mass spectrometry) and 386,449 single nucleotide polymorphisms (SNPs). Our sample consisted of 1,829 participants randomly selected from the Sister Study, a cohort of women who had a sister with breast cancer but had never had breast cancer themselves. 19,741 SNPs were associated with 25(OH)D (*p* < 0.05). We re-assessed these hits in an independent sample of 1,534 participants who later developed breast cancer. After pooling, 32 SNPs had genome-wide significant associations (*p* < 5 × 10^−8^). These were located in or near *GC*, the vitamin D binding protein, or *CYP2R1*, a cytochrome P450 enzyme that hydroxylates vitamin D to form 25(OH)D. The top hit was rs4588, a missense *GC* polymorphism associated with a 3.5 ng/mL decrease in 25(OH)D per copy of the minor allele (95% confidence interval [CI]: −4.1, −3.0; *p* = 4.5 × 10^−38^). The strongest SNP near *CYP2R1* was rs12794714, a synonymous variant (*p* = 3.8 × 10^−12^; β = 1.8 ng/mL decrease in 25(OH)D per minor allele [CI: −2.2, −1.3]). Serum 25(OH)D concentrations from samples collected from some participants 3–10 years after baseline (811 cases, 780 non-cases) were also strongly associated with both loci. These findings augment our understanding of genetic influences on 25(OH)D and the possible role of vitamin D binding proteins and cytochrome P450 enzymes in determining measured levels. These results may help to identify individuals genetically predisposed to vitamin D insufficiency.

## Introduction

While randomized clinical trials of have failed to establish direct links between vitamin D supplementation and health (Bjelakovic et al., 2011, 2014; Avenell et al., 2014), observational studies have demonstrated that high levels of 25-hydroxyvitamin D [25(OH)D], a vitamin D biomarker that can be measured in blood, are associated with lower mortality (Garland et al., 2014) and reduced risk of many chronic diseases, including heart disease and some cancers (Gandini et al., 2011; Autier et al., 2014; Zhang et al., 2017). Determinants of 25(OH)D include ultraviolet-B radiation, dietary supplements, and certain foods, including fish and fortified dairy products. Genetic factors are also thought to play a role in determining blood concentrations.

Vitamin D sufficiency is typically assessed by measuring serum or plasma concentrations of 25(OH)D, which is a stable precursor to the active form of vitamin D, 1,25(OH)_2_D. The Institute of Medicine guidelines suggest that 25(OH)D concentrations above 20 ng/mL are sufficient for bone health (Ross et al., 2011), but higher levels may provide additional health benefits (Garland and Gorham, 2016; Durazo-Arvizu et al., 2017; O'Brien et al., 2017b; Zhang et al., 2017). Previous genome-wide association studies (GWAS) have identified several single nucleotide polymorphisms (SNPs) associated with 25(OH)D concentrations. To date, eight such GWAS have been published (Benjamin et al., 2007; Ahn et al., 2010; Engelman et al., 2010; Wang et al., 2010; Lasky-Su et al., 2012; Anderson et al., 2014; Sapkota et al., 2016; Jiang et al., 2018), identifying a total of 17 SNPs in 7 chromosomal locations (in or near *GC, NADSYN1/DHCR7, CYP2R1/RRAS2/PDE3B, CYP24A1, SSTR4/FOXA2, AMDHD1*, and *SEC23A*) with association *p*-values < 5 × 10^−8^ (PheGen I: Phenotype-Genotype Integrator-National Center for Biotechnology Information, 2014^1^; MacArthur et al., 2017).

Here, we introduce the first vitamin D GWAS to use the current gold-standard measure of 25(OH)D, liquid chromatography/tandem mass spectrometry (LC/MS). LC/MS has been shown to outperform other commonly-utilized methods (Farrell et al., 2012) and is capable of measuring concentrations of 3-epi-25(OH)D_3_, which is thought to play a similar biological role to 25(OH)D_3_ (Cashman et al., 2014). We conducted this work using serum collected from a random sample of women participating in a large prospective cohort study. We re-assessed the top SNPs in an independent set of serum samples from women in the same cohort who later developed breast cancer, and again in serum samples collected from some of the same cases (post-diagnosis) and non-cases 3–10 years after baseline. We also performed haplotype analyses of key genetic regions.

## Materials and methods

This work was conducted using data from the Sister Study, a prospective cohort of 50,884 women who had a full or half-sister with a history of breast cancer, but who had never had breast cancer themselves at the time they enrolled in the study. Participants aged 35–74 and living in the United States or Puerto Rico joined the study between 2003 and 2009. They were visited in their homes by a trained examiner, who obtained written informed consent and collected the blood samples needed for 25(OH)D and genotype analyses. A subset of participants, including some who had developed breast cancer and some who had not, were asked to provide a second blood sample and other biospecimens in 2013–2014, 3–10 years after baseline. Further details on the study protocol, which also included extensive questionnaires and additional biospecimens, are available elsewhere (Sandler et al., 2017). Approval and oversight for the Sister Study is provided by the Institutional Review Boards of the National Institute of Environmental Health Sciences and the Copernicus group. Analyses were completed using data release 4.1 (July 2014).

Participants for the vitamin D and genetics sub-studies were selected using a case-cohort design that included a random sample from the full cohort (*n* = 1,829, including 67 women who went on to develop breast cancer) and all remaining women diagnosed with invasive breast cancer or ductal carcinoma *in situ* within 5 years of their baseline blood draw (*n* = 1,534 additional cases, for 1,601 total cases) (O'Brien et al., 2017b). This included 28 pairs of sisters, who were treated as independent observations despite their genetic similarities [between-sister *R*^2^ for 25(OH)D levels = 0.03]. Blood samples from these women were assayed for 533,631 SNPs using the Infinium OncoArray genotyping panel (Illumina Inc.) (Amos et al., 2016). This panel includes a full GWAS backbone, as well as ancestry informative markers (AIMs) and genes presumably or possibly linked to cancer or cancer-related factors. Serum samples were assessed for 25(OH)D concentrations using LC/MS at Heartland Assays, Inc. Measured concentrations were adjusted for batch effects and season of blood draw and thus approximate average annual 25(OH)D. Further details on both the SNP and 25(OH)D analysis can be found elsewhere (Amos et al., 2016; O'Brien et al., 2017a,b).

After excluding SNPs that did not meet quality control standards (*n* = 41,664, as described previously, Amos et al., 2016) or that had a minor allele frequency (MAF) less than 2% in the sub-cohort (*n* = 105,518), 386,449 SNPs remained. We calculated Hardy-Weinberg equilibrium *p*-values and examined our top hits for evidence of disequilibrium, but did not exclude SNPs based on these results. We regressed 25(OH)D on the number of copies of the minor allele for each of these SNPs using linear least-squares. The values of 25(OH)D looked normally distributed. We adjusted each model for age at blood draw (in years), self-reported race/ethnicity [Non-Hispanic White (*n* = 1,576), Black (*n* = 134), Hispanic (*n* = 81), or other (*n* = 38)] and genetic ancestry (proportion CEU, YRI, or CHB) (O'Brien et al., 2017a) and calculated the genomic control inflation factor (λ) to test for evidence of uncontrolled confounding due to population stratification. We also conducted sensitivity analyses adjusting for estimated total vitamin D intake at baseline (dietary plus supplement) and hours spent outdoors per year, as these both contribute to variations in measured 25(OH)D concentrations. Analyses were conducted using SAS (v9.3), R (v3.2.1), or PLINK (v1.07). Locus plots were made using LocusZoom (Pruim et al., 2011).

Primary analyses used the baseline 25(OH)D measurements from the randomly selected sub-cohort. For replication analyses, we also analyzed associations in the set of women who later developed breast cancer, assessing the relationship between 25(OH)D and any SNPs for which the *p*-value had been < 0.05 in the sub-cohort. We excluded the 67 cases selected into the sub-cohort. In analyses pooling the sub-cohort and cases we defined genome-wide significance as *p* < 5 × 10^−8^. The pooled analysis included additional adjustment for future case status.

We also examined the association between these SNPs and 25(OH)D concentrations in blood samples collected 3–10 years after enrollment. In 2013, a total of 3,762 women who were originally selected for participation in the case-cohort sample were asked to provide secondary biospecimens. We collected second blood samples for 1,227 women who had been diagnosed with breast cancer while on study [assaying 811 for 25(OH)D] and 1,203 who had remained breast cancer-free [assaying 780 for 25(OH)D]. Measurements were based on LC/MS (Heartland Assays, Inc.).

We also conducted haplotype analyses of the top two regions using baseline data from the sub-cohort and cases. These were conducted separately in Non-Hispanic whites and African-Americans due to concerns about population stratification and racial differences in linkage disequilibrium (Stram and Seshan, 2012). We used expectation-maximization software (“hapassoc” package in R; Burkett et al., 2004, 2006) to estimate haplotype frequencies and the association between 25(OH)D and each copy of the index haplotype, relative to the most common haplotype in non-Hispanic whites. We assessed all haplotypes with estimated frequency of at least 2%, pooling the rare haplotypes into a single category. Models were adjusted for age at blood draw, ancestry, and future case status.

## Results

Women in the sub-cohort were 55.3 years of age, on average, when they joined the study and had average serum 25(OH)D concentrations of 31.8 ng/mL (Table 1). Most participants were non-Hispanic white (86%). Women who later developed breast cancer were 57.4 years of age at baseline and had average 25(OH)D concentration of 31.0 ng/mL. On average, 7.8 years passed between baseline and follow-up blood collection. At that time, serum 25(OH)D concentrations were 40.4 ng/mL in non-cases and 43.5 ng/mL in post-diagnosis cases. Non-Hispanic white women comprised 89% the follow-up group.

**Table 1:** Description of study sample.

	**Baseline**		**Second blood draw 3–10 years after baseline (“Sisters Changing Lives”)**	
	**Sub-cohort (*n* = 1,829)^b^**	**Cases (*n* = 1,534)^c^^,^^d^**	**Non-cases (*n* = 780)**	**Cases (*n* = 811)^d^**
Age at blood draw; mean (std)	55.3 (8.9)	57.4 (8.9)	63.1 (8.7)	65.5 (8.8)
25(OH)D (ng/mL)^a^; mean (std)	31.8 (10.5)	31.0 (10.1)	40.4 (13.7)	43.5 (14.3)
**Self-reported Race/Ethnicity;** ***n*** **(%)**				
Non-hispanic white	1576 (86)	1307 (85)	693 (89)	722 (89)
African-American	134 (7)	120 (8)	46 (6)	38 (5)
Hispanic	81 (4)	59 (4)	27 (3)	27 (3)
Other	38 (2)	47 (3)	14 (2)	23 (3)

a *Adjusted for batch and season of blood draw*.

b *Includes 67 women who went on to develop breast cancer*.

c *Excludes cases selected as part of the random sub-cohort*.

d *1 case missing self-reported race/ethnicity*.

**Figure 1:**
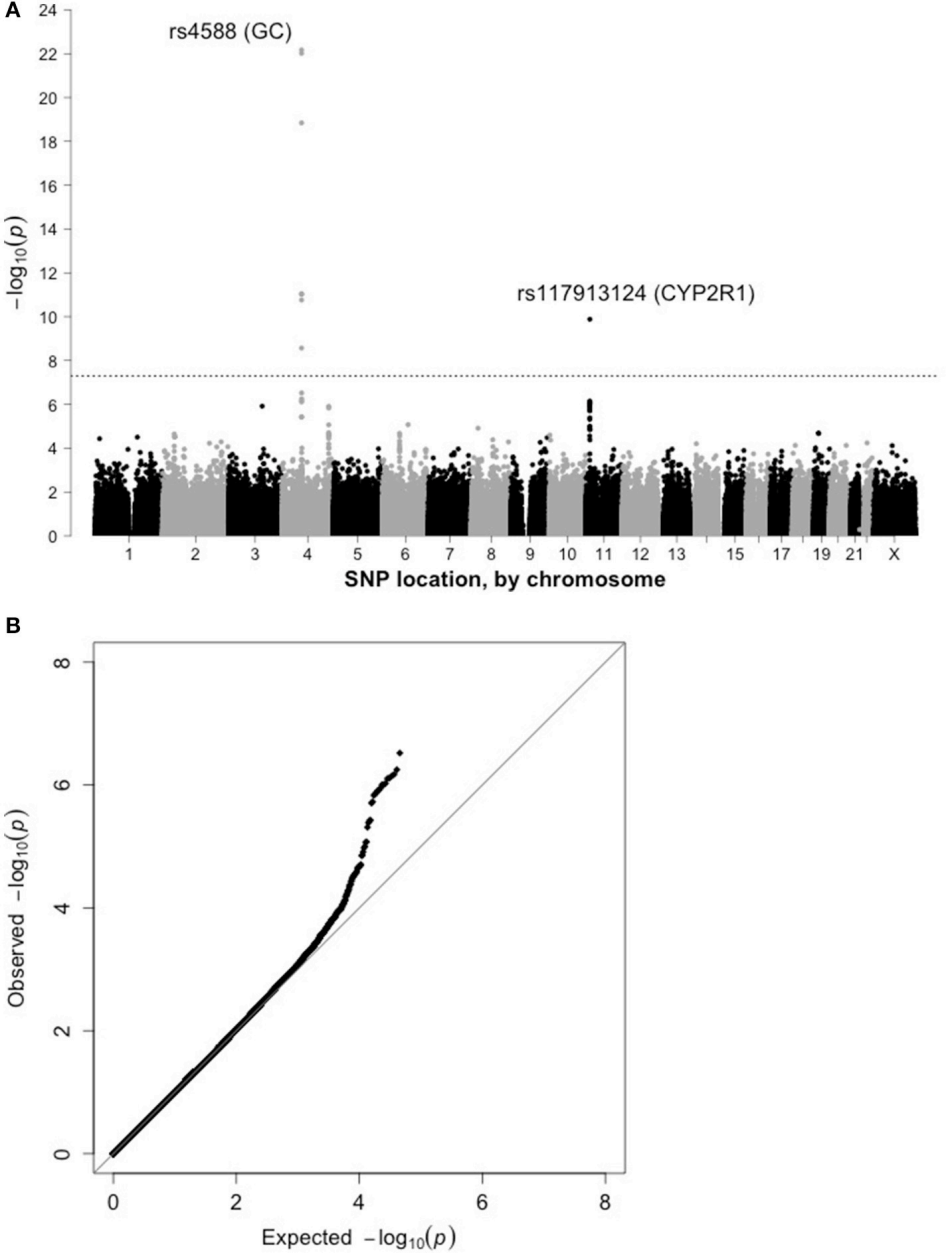
**(A)** Manhattan plot for 25(OH)D in the sub-cohort (*n* = 1,829). **(B)** Quantile-quantile plot for 25(OH)D in the sub-cohort (*n* = 1,829).

Results from preliminary analyses conducted within the sub-cohort are shown as a Manhattan plot (Figure 1A) and quantile-quantile plot (Figure 1B). There was no evidence of residual confounding due to population stratification (λ = 1.007). 19,741 SNPs were associated with 25(OH)D at *p* < 0.05. The top hit was rs4588 (*p* = 6.8 × 10^−23^; Table 2), located in the vitamin D binding protein (VDBP) gene (*GC*) on chromosome 4. SNPs in or near *CYP2R1*, which encodes a cytochrome P450 vitamin D hydroxylase, also showed evidence of an association with 25(OH)D, with rs117913124 having the lowest *p*-value in the sub-cohort (*p* = 1.3 × 10^−10^).

**Table 2:** Single nucleotide polymorphism associated with serum 25(OH)D levels at *p* < 5 × 10^−8^ in the Sister Study (2003–2009).

**SNP-rare allele**	**Location^a^**	**MAF, sub-cohort**	**HWE *p*-value, subcohort**	***P*-value, sub-cohort**	***P*-value, cases**	**β^b^ (95% CI), pooled sample^c^**	***P*-value, pooled sample^c^**	***P*-value in follow-up study^c^^,^^d^**
**CHROMOSOME 4**, ***GC***								
**rs4588-A**	**72618323**	**0.27**	**0.55**	**6.8 × 10^−23^**	**8.9 × 10^−17^**	**−3.5 (−4.1, −3.0)**	**4.5 × 10^−38^**	**5.8 × 10^−10^**
rs2282679-C	72608383	0.26	0.28	9.6 × 10^−23^	3.1 × 10^−16^	−3.5 (−4.0, −3.0)	1.9 × 10^−37^	9.0 × 10^−10^
rs1155563-G	72643488	0.27	0.05	1.4 × 10^−19^	2.9 × 10^−11^	−3.0 (−3.5, −2.5)	9.6 × 10^−29^	6.6 × 10^−7^
rs705120-A	72614140	0.42	0.60	9.5 × 10^−12^	1.6 × 10^−11^	−2.4 (−2.8, −1.9)	4.5 × 10^−22^	6.2 × 10^−5^
rs2201124-A	72597009	0.31	0.70	9.0 × 10^−12^	3.5 × 10^−7^	−2.2 (−2.8, −1.7)	3.4 × 10^−17^	3.2 × 10^−6^
rs4694105-A	72592214	0.31	0.62	1.7 × 10^−11^	2.2 × 10^−7^	−2.2 (−2.8, −1.7)	3.5 × 10^−17^	4.7 × 10^−6^
rs1526692-G	72578724	0.40	0.31	2.7 × 10^−9^	6.8 × 10^−8^	−2.0 (−2.5, −1.5)	8.8 × 10^−16^	1.9 × 10^−5^
rs6837549-A	72596821	0.49	0.32	6.7 × 10^−7^	1.1 × 10^−6^	1.7 (1.2, 2.2)	4.6 × 10^−12^	0.003
rs13113067-A	72739098	0.35	0.26	5.6 × 10^−7^	0.001	−1.5 (−2.0, −1.0)	3.8 × 10^−9^	0.14
rs12639968-A	72712872	0.18	0.53	7.7 × 10^−7^	0.001	−1.8 (−2.4, −1.2)	7.7 × 10^−9^	0.01
rs962227-A	72707517	0.29	0.17	3.0 × 10^−7^	0.005	−1.5 (−2.1, −1.0)	1.1 × 10^−8^	0.03
rs10033936-G	72743474	0.25	0.26	3.8 × 10^−6^	7.5 × 10^−4^	−1.6 (−2.2, −1.0)	2.2 × 10^−8^	0.09
rs12512631-G	72601331	0.35	0.72	3.7 × 10^−6^	0.004	1.4 (0.9, 1.9)	3.8 × 10^−8^	0.003
**CHROMOSOME 11, *CYP2R1***								
**rs12794714-A**	**14913575**	**0.42**	**0.18**	**7.9 × 10^−7^**	**9.4 × 10^−7^**	**−1.8 (−2.2, −1.3)**	**3.8 × 10^−12^**	**4.1 × 10^−4^**
rs201473898-A	14893704	0.42	0.23	9.4 × 10^−7^	1.2 × 10^−6^	−1.7 (−2.2, −1.2)	6.5 × 10^−12^	3.5 × 10^−4^
rs11023227-G	14459087	0.36	0.27	7.1 × 10^−7^	2.2 × 10^−6^	−1.7 (−2.2, −1.2)	1.5 × 10^−11^	0.02
rs10832275-C	14478224	0.36	0.21	1.3 × 10^−6^	1.6 × 10^−6^	−1.7 (−2.2, −1.2)	1.6 × 10^−11^	0.02
rs7121171-G	14446420	0.36	0.16	9.9 × 10^−7^	2.3 × 10^−6^	−1.7 (−2.2, −1.2)	1.6 × 10^−11^	0.02
rs12295888-G	14450531	0.36	0.21	9.9 × 10^−7^	2.0 × 10^−6^	−1.7 (−2.2, −1.2)	1.7 × 10^−11^	0.02
rs72261784-D	14446060	0.36	0.22	1.4 × 10^−6^	2.2 × 10^−6^	−1.7 (−2.2, −1.2)	2.5 × 10^−11^	0.02
rs11023223-G	14457112	0.36	0.19	1.1 × 10^−6^	2.6 × 10^−6^	−1.7 (−2.2, −1.2)	2.5 × 10^−11^	0.02
rs10832294-A	14747427	0.41	0.27	4.1 × 10^−6^	1.5 × 10^−6^	−1.7 (−2.2, −1.2)	4.2 × 10^−11^	3.8 × 10^−4^
rs10832268-A	14465068	0.36	0.19	1.9 × 10^−6^	3.0 × 10^−6^	−1.7 (−2.2, −1.2)	4.7 × 10^−11^	0.01
rs11023332-G	14784110	0.42	0.31	1.0 × 10^−5^	6.6 × 10^−7^	−1.7 (−2.2, −1.2)	5.1 × 10^−11^	7.5 × 10^−4^
rs10832269-A	14465069	0.36	0.19	2.0 × 10^−6^	3.3 × 10^−6^	−1.7 (−2.1, −1.2)	5.6 × 10^−11^	0.01
rs11023246-G	14536956	0.36	0.29	4.9 × 10^−6^	1.7 × 10^−6^	−1.7 (−2.2, −1.2)	6.2 × 10^−11^	0.03
rs117913124-A	14900931	0.02	1.00	1.3 × 10^−10^	0.01	−5.1 (−6.7, −3.6)	1.2 × 10^−10^	4.2 × 10^−6^
rs2305305-A	14540942	0.36	0.26	1.1 × 10^−5^	1.8 × 10^−6^	−1.6 (−2.1, −1.1)	1.5 × 10^−10^	0.03
rs10766188-G	14660826	0.34	0.38	1.8 × 10^−4^	3.9 × 10^−6^	−1.5 (−2.0, −1.0)	6.1 × 10^−9^	0.003
rs1993116-A	14910234	0.38	0.69	1.4 × 10^−5^	1.3 × 10^−4^	1.4 (0.9, 1.9)	9.7 × 10^−9^	6.3 × 10^−4^
rs10741657-A	14914878	0.37	0.73	4.2 × 10^−5^	2.2 × 10^−4^	1.4 (0.9, 1.9)	4.7 × 10^−8^	0.002
rs11023203-A	14409815	0.33	0.34	2.8 × 10^−5^	3.3 × 10^−4^	−1.4 (−1.9, −0.9)	4.9 × 10^−8^	0.03

*All models adjusted for age at blood draw, self-reported race/ethnicity, and estimated ancestry proportions; MAF, minor allele frequency; HWE, Hardy-Weinberg equilibrium*.

a *GRch37/hg19*.

b *Change in 25(OH)D (in ng/mL) per copy of the minor allele*.

c *Adjusting for case status*.

d *Serum 25(OH)D levels measured in blood collected 3–10 years after baseline for the “Sisters Changing Lives” follow-up study*.

*Bold values indicate SNP with lowest p-value for the region (based on pooled sample)*.

**Figure 2:**
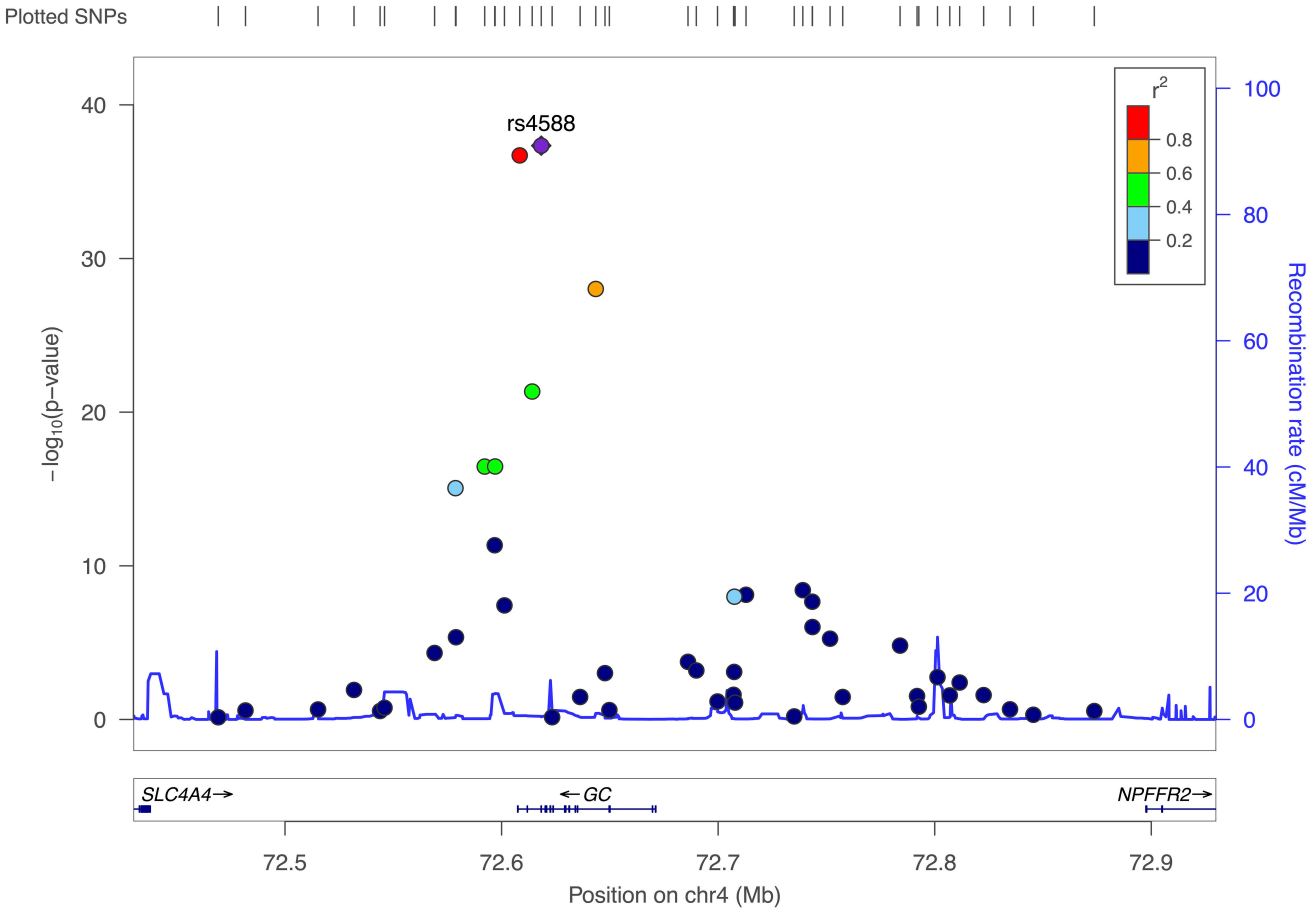
Fine-mapping of region surrounding rs4588 (chromosome 4), all participants.

When we re-assessed the top 19,741 SNPs in the independent sample of women who later developed breast cancer, 1,121 were statistically-significant at *p* < 0.05. For the pooled sample, which included both cases and sub-cohort members, we identified 32 SNPs in two regions that were associated with 25(OH)D at *p* < 5 × 10^−8^ (Table 2). The top SNP for the *GC* region was again rs4588 (*p* = 4.5 × 10^−38^), with each copy of the variant A allele associated with an estimated 3.5 ng/mL decrease in 25(OH)D (95% confidence interval [CI]: −4.1, −3.0). This SNP also had a strong association in the smaller follow-up sample (*p* = 5.8 × 10^−10^). Twelve other SNPs in the region surrounding rs4588 were also strongly associated with 25(OH)D in the pooled baseline sample (Table 2 and Figure 2) and, with a few exceptions, also in the follow-up sample.

**Figure 3:**
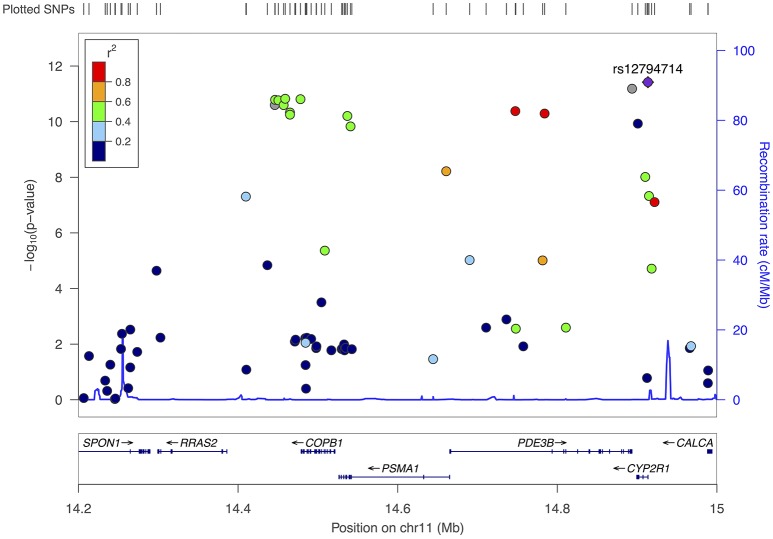
Fine-mapping plot for the region surrounding rs12794714 (chromosome 11), all participants.

A more common *CYP2R1* SNP, rs12794714, replaced rs117913124 as the top hit in the region for the pooled sample (*p* = 3.8 × 10^−12^ and MAF = 0.42 for rs12794714 vs. *p* = 1.2 × 10^−10^ and MAF = 0.02 for rs117913124), with each copy of the A allele associated with a 1.8 ng/mL decrease in 25(OH)D (95% CI: −2.2, −1.3). Seventeen other SNPs in this region also met criteria for genome-wide statistical significance in the pooled sample (Table 2). Though most of these were in moderate to high linkage disequilibrium with rs12794714, they spanned several gene regions, including *COPB1, PSMA1, PDE3B*, and *RRAS2* (Figure 3). A second peak appeared ~454 kb away from rs12794714 at rs11023227 in *COPB1*, but the signal was not independent (original *p* = 1.5 × 10^−11^; *p* = 0.007 after adjusting for rs12794714; *r*^2^ = 0.33). All 19 of the genome-wide significant hits in this region were also associated with 25(OH)D concentrations from the follow-up visit (all *p* ≤ 0.03). None of the genome-wide significant hits showed evidence of Hardy-Weinberg disequilibrium.

Fifteen other SNPs that had false discovery rate *q*-values < 0.10 in the pooled sample are described in Supplementary Table 1. Three were from the chromosome 4 locus and four from the chromosome 11 locus (one on *COPB1* and two on *PDE3B*). The remaining eight were independent signals, only two of which represented known genes (rs4951247 on *ELK4* and rs360157 on *MYO9B*). The associations between these SNPs and 25(OH)D measured during follow-up were mostly consistent with those observed for baseline 25(OH)D.

When we additionally adjusted for both estimated vitamin D intake at baseline (measured using a food frequency questionnaire plus self-reported supplement use) and self-reported average hours spent outdoors per year, the results were very similar, with all but one of 32 SNPs reported here showing genome-wide significant associations (Supplementary Table 2). While the rankings shuffled around for the chromosome 11 locus, the only notable change was that while rs117913124 was the top ranked SNP for both the sub-cohort specific and pooled analysis, rs12794714 dropped down to 11th with the additional adjustments. There was little change to any of the effect estimates or *p*-values.

For haplotype analyses (Table 3), which were based on the pooled baseline sample, we selected SNPs with very small *p*-values (*p* < 1 × 10^−10^ for the chromosome 4 region and *p* < 5 × 10^−8^ for the chromosome 11 region) that were not in high linkage disequilibrium with any other, more strongly associated SNP (*r*^2^ < 0.80 in our sample). We excluded rs117913124 due to its low MAF. This resulted in a 6-SNP set including rs4588 and a 5-SNP set including rs12794714. For non-Hispanic whites, there were seven common (frequency >2%) haplotypes for both chromosomal sets. For chromosome 4, each copy of the “GCAAAG” haplotype (frequency = 21%) was associated with a 3.9 ng/mL decrease in 25(OH)D (95% CI: −4.6, −3.2; *p* = 9.8 × 10^−30^) in non-Hispanic whites, relative to the most common haplotype (AAGCCA, frequency = 43%). In African-Americans, only “GCACCA” (frequency = 9%) was significantly associated with 25(OH)D levels, with an estimated 4.6 ng/mL increase in 25(OH)D (95% CI: 1.2, 8.0; *p* = 0.008), per copy, relative to “AAGCCA” (frequency = 17%). For chromosome 11, “AGGGA” (frequency = 26%) was associated with an estimated 2.1 ng/mL decrease per copy in non-Hispanic whites (95% CI: −2.8, −1.5; *p* = 6.1 × 10^−10^), relative to the most common “GAAAG” haplotype (frequency = 34%). Haplotype distributions for the chromosome 11 locus were very different for African-Americans, with “GAAGG” being the most common haplotype (frequency = 53%) and no haplotypes showing a statistically-significant difference in 25(OH)D concentrations compared to “GAAAG” (frequency = 26%). Correlation matrices for SNPs in both of these regions are included as Supplementary Figures 1, 2.

**Table 3:** Haplotype analysis.

**Haplotype**	**Non-Hispanic whites (*n* = 2,883)**			**African-Americans (*n* = 254)**		
	**Haplotype frequency**	**β^a^ (95% CI), pooled sample^e^**	***P*-value, pooled sample^e^**	**Haplotype frequency**	**β^a^ (95% CI), pooled sample^e^**	***P*-value, pooled sample^e^**
**CHROMOSOME 4 (*****GC*****):** *rs1526692, rs6837549, rs2201124, rs705120, **rs4588**, rs1155563*						
AAGC**C**A^a^	0.43	0.0		0.17	0.0	
GCAA**A**G^b^	0.21	−3.9 (−4.6, −3.2)	9.8 × 10^−30^	0.05	2.7 (−1.8, 7.1)	0.24
ACGA**C**A^c^	0.07	−1.0 (−2.0, 0.0)	0.05	0.10	1.1 (−2.5, 4.7)	0.54
GCAC**C**A^a^	0.07	−0.7 (−1.7, 0.3)	0.17	0.09	4.6 (1.2, 8.0)	8.5 × 10^−3^
GCGA**C**A^c^	0.07	−1.3 (−2.4, −0.3)	0.01	0.12	2.1 (−1.2, 5.5)	0.21
AAGA**A**G^b^	0.03	−4.6 (−6.2, −3.1)	3.2 × 10^−9^	–	–	–
ACGC**C**A^a^	0.03	−0.7 (−2.4, 0.9)	0.40	0.16	−1.0 (−5.2, 2.1)	0.52
GAGC**C**A^a^	–	–	–	0.10	−1.4 (−5.1, 2.3)	0.47
GAGA**C**A^c^	–	–	–	0.06	−1.9 (−6.5, 2.7)	0.43
AAGA**C**A^c^	–	–	–	0.04	1.0 (−4.9, 6.9)	0.74
Rare pooled^d^	0.08	−2.3 (−3.3, −1.3)	5.4 × 10^−6^	0.11	−1.9 (−5.2, 1.4)	0.26
**CHROMOSOME 11 (*****CYP2R1*****):** *rs11023203, rs11023227, rs10766188, rs1993116, **rs12794714***						
GAAA**G**	0.34	0.0		0.26	0.0	
AGGG**A**	0.26	−2.1 (−2.8, −1.5)	6.1 × 10^−10^	0.09	−2.4 (−5.8, 1.0)	0.17
GAAG**G**	0.15	−0.7 (−1.5, 0.1)	0.07	0.53	−1.8 (−7.2, 3.7)	0.53
GAAG**A**	0.06	−1.4 (−2.5, −0.2)	0.02	0.03	−1.6 (−3.9, 0.6)	0.15
GGGG**A**	0.05	−2.0 (−3.2, −0.8)	1.5 × 10^−3^	–	–	–
GAGG**A**	0.03	−1.8 (−3.3, −0.3)	0.02	–	–	–
AGAG**A**	0.03	−2.9 (−4.6, −1.3)	4.0 × 10^−4^	–	–	–
Rare pooled^d^	0.08	−1.0 (−2.1, 0.0)	0.05	0.11	−0.8 (−3.9, 2.2)	0.59

*All models adjusted for age at blood draw, estimated ancestry proportions, and breast cancer case status*.

a *Corresponds to the “Gc1s” vitamin D binding protein variant in Non-Hispanic whites (based on genotypes for rs705120 and rs4588)*.

b *Corresponds to the “Gc2” vitamin D binding protein variant in Non-Hispanic whites (based on genotypes for rs705120 and rs4588)*.

c *Corresponds to the “Gc1f” vitamin D binding protein variant in Non-Hispanic whites (based on genotypes for rs705120 and rs4588)*.

d *Rare haplotypes (frequency < 2%) pooled together*.

e *Change in 25(OH)D (in ng/mL) per copy of the index haplotype relative to the most common haplotype (“AAGCCA” for chromosome 4, “GAAAG” for chromosome 11), controlling for all other haplotypes*.

*Bold values indicate SNP with lowest independently measured p-value (based on pooled sample)*.

## Discussion

In this GWAS of vitamin D serum levels, we identified two regions strongly associated with serum 25(OH)D—one on chromosome 4 surrounding the *GC* gene and the second on chromosome 11 including SNPs from *CYP2R1, COPB1, PSMA1*, and *PDE3B*. The identified loci replicated in an independent sample of women selected because they later developed breast cancer and the SNPs were also strongly associated with 25(OH)D concentrations measured in second blood samples from the same participants collected 3–10 years after baseline. To our knowledge, this was the first GWAS to use the gold-standard LC/MS methods to measure total 25(OH)D and the first to examine haplotypes.

The *GC* gene encodes the VDBP, a member of the albumin family that stores and transports both 25(OH)D and the active form of vitamin D, 1,25(OH)_2_D (Speeckaert et al., 2006). It seems quite plausible that a variant that affects VDBP could directly impact measured serum 25(OH)D. *CYP2R1* polymorphisms also have the capacity to directly impact 25(OH)D concentrations, as this gene encodes a cytochrome P450 enzyme responsible for hydroxylating vitamin D and converting it to 25(OH)D (Shinkyo et al., 2004).

Our findings augment the results of previous GWAS. Those that reported hits in *GC* (Ahn et al., 2010; Wang et al., 2010; Lasky-Su et al., 2012; Anderson et al., 2014; Jiang et al., 2018) observed the smallest *p*-values for rs1155563, rs17467825, rs2282679, and rs3755967. We did not assess rs17467825 or rs3755967, but rs2282679 and rs1155563 had the second and third smallest *p*-values in our study. However, they both are synonymous substitutions, while rs4588 is a missense substitution in an exon (exon 12; amino acid change Thr to Lys at position 436). Therefore, given their high correlations with rs4588 (*r*^2^ = 0.97 and 0.77, respectively), these previously identified SNPs may just be tags for rs4588, which may be a variant that causally impacts 25(OH)D concentrations. This possibility is supported by a number of candidate gene studies that have reported strong associations between rs4588 and measured 25(OH)D (Engelman et al., 2008; Lu et al., 2012; Perna et al., 2013; Robien et al., 2013; Li et al., 2014; Nissen et al., 2014). Of note, rs4588 was not associated with gene expression in quantitative trait loci analyses, but five other nearby genome-wide significant *GC* SNPs (rs1155563, rs13113067, rs12639968, rs962227, and rs10033936) were associated with expression of VDBP in stomach tissue^2^.

Another putative causal variant is rs7041, which also results in a missense substitution in exon 12 (amino acid change Asp to Glu, position 432). We did not measure this SNP, but it is highly correlated with rs705120 in whites (*r*^2^ = 0.97 in the CEU sample of 1,000 genomes) (Johnson et al., 2008; 1000 Genomes Project Consortium et al., 2015), and rs705120 had a *p*-value of 9.5 × 10^−12^ in our sample. Together, the genotypes for rs4588 and rs705120/rs7041 determine individual's VDBP variants (“AA” = Gc2, “AC” = Gc1f, or “CC” = Gc1s) (Powe et al., 2013). The combination of these variants determine an individual's phenotype, where each phenotype has a different glycosylation pattern and binding affinity (Braun et al., 1992; Abbas et al., 2008; Powe et al., 2013). In our sample, carriers of the Gc2-coding haplotypes had the lowest 25(OH)D concentrations (Table 3). This is consistent with the results of one previous study, which showed that individuals homozygous for Gc2-coding haplotypes (Gc2/Gc2 phenotype) had significantly lower serum concentrations of 25(OH)D than those with Gc1S/Gc1S, Gc1S/Gc1F, or Gc1s/Gc2 phenotypes (Sollid et al., 2016). The same study reported that individuals with the Gc2/Gc2 phenotype had lower VDBP than all other phenotypes.

However, the links between rs4588, rs705120/rs7041, *GC* haplotypes, and concentrations of VDBP and 25(OH)D remain uncertain, as other studies, including some with large samples of African-Americans, have not observed these same associations (Powe et al., 2013; Batai et al., 2014; Denburg et al., 2016; Yao et al., 2017). The role of the other SNPs included in this haplotype analysis is also unclear, though we note that any haplotypes that contained “A” alleles for both rs4588 and rs705120 also contained the “G” allele for rs1155563, a synonymous polymorphism identified in a previous GWAS (Anderson et al., 2014).

In our sample, African-Americans were less likely to carry the minor allele at rs4588 (MAF = 0.11 vs. 0.28 in non-Hispanic whites; Supplementary Table 3) and the SNP was not associated with 25(OH)D in African-Americans, though our sample size was quite small (data not shown). In general, most of the GWAS and candidate gene studies that observed positive associations between 25(OH)D and SNPs in *GC* were conducted in populations of European or Chinese descent (Engelman et al., 2008, 2010; Ahn et al., 2010; Wang et al., 2010; Jorde et al., 2012; Lasky-Su et al., 2012; Lu et al., 2012; Perna et al., 2013; Robien et al., 2013; Zhang et al., 2013; Anderson et al., 2014; Batai et al., 2014; Li et al., 2014; Nissen et al., 2014; Clendenen et al., 2015), with less consistent results seen for African-American populations (Engelman et al., 2008; Batai et al., 2014; Yao et al., 2017).

As rs705120 is not correlated with rs7041 in African-Americans (*r*^2^ = 0.10 in YRI sample of 1,000 genomes), we cannot determine the VDBP variants of our African-American participants, but prior studies have reported that VDBP variant distributions differ markedly by race, with Gc1F being much more common in African-Americans than whites (Powe et al., 2013; Denburg et al., 2016). Our inability to capture phenotype-relevant haplotypes in African-Americans may explain why we observed no clear associations between *GC* haplotypes and 25(OH)D concentrations in this group. The positive association between 25(OH)D and “GCACCA” vs. the referent “AAGCCA” haplotype may indicate a more influential role of the first three SNPs (rs1526692, rs6837549, rs2201124) or other nearby correlated SNPs in African-American women, but we again note that our African-American-specific results are based on very small numbers. Larger studies of African-American are needed to help disentangle these complicated relationships.

The relationship between the SNPs on chromosome 11 and 25(OH)D is also complex. Our top hit for the region, rs12794714, is a synonymous substitution in exon 1 of *CYP2R1*. The rare SNP with the strongest association in the sub-cohort, rs117913124 (MAF = 0.02), also results in a synonymous substitution in *CYP2R1* (exon 4) and in a recent whole genome sequencing analysis (Manousaki et al., 2017), this variant was strongly associated with vitamin D even after adjusting for more common, previously-identified SNPs in the same region. Earlier GWAS for serum 25(OH)D identified 5 genome-wide significant SNPs on chromosome 11 near *CYP2R1* (rs10741657, rs2060793, rs11023332, rs12287212, rs1007392) (Ahn et al., 2010; Wang et al., 2010; Anderson et al., 2014; Jiang et al., 2018). Of these previous GWAS hits, we assessed rs10741657 and rs11023332 (*p* = 4.9 × 10^−8^ and *p* = 1.5 × 10^−11^, respectively, in the pooled sample). The former is close to rs12794714 (1.3 kb upstream), but is intergenic and the two are only loosely correlated (*r*^2^ = 0.40 in our sample). The latter is further away from rs12794714 (129 kb downstream), but is more strongly correlated (*r*^2^ = 0.90) and results in a silent substitution in *PDE3B*. None of the other previously identified hits is an obvious candidate for a causal association: rs1007392 is an intron variant of *PDE3B*, rs2060793 is located upstream of *CYP2R1*, and rs12287212 is intergenic. Though many candidate gene studies have assessed the association between 25(OH)D and rs12794714 or other highly correlated SNPs, there is no clear consensus as to the likely causal variant(s) (Ramos-Lopez et al., 2007; Zhang et al., 2012, 2013; Robien et al., 2013; Batai et al., 2014; Ordóñez-Mena et al., 2016). Previously documented ancestral heterogeneity in allele frequencies and measured associations contribute additional uncertainty (Batai et al., 2014; Elkum et al., 2014). Assessments of expression quantitative trait loci showed that the genome-wide significant SNPs in this region were only associated with expression of genes in the direct vicinity (*CYP2R1, RRAS2, COPB1, CALCB*, and *PDE3B*)^2^.

We did not see genome-wide significant association between 25(OH)D and any of the SNPs in *DHCR7/NADSYN1* or *SSTR4/FOXA2* identified in previous GWAS (Wang et al., 2010; Sapkota et al., 2016; Jiang et al., 2018), though one of the *DHCR7/NADSYN1* hits, rs12785878, had a very low *p*-value (0.0007 in the sub-cohort and 5.9 × 10^−5^ in the pooled sample) with the minor allele associated with decreased 25(OH)D in both their report (Wang et al., 2010) and our sample (β = −1.1 ng/mL per allele, 95% CI: −1.5, −0.6 for our study). None of the previously reported *SSTR4/FOXA2* SNPs showed evidence of an association in our data (all uncorrected *p* > 0.3), though we note that these SNPs were originally identified in a sample of Punjabi Sikhs (Sapkota et al., 2016). As none of the SNPs identified in a recent genome-wide meta-analysis of men and women of European descent (Jiang et al., 2018) were directly genotyped in our sample (rs17216707 in *CYP24A1*, rs10745742 in *AMDHD1*, or rs8018720 in *SEC23A*), we looked for signals in nearby SNPs, finding none with low *p*-values (all >0.05). This failure to replicate could be due to chance, lack of power in our study, which was much smaller than the meta-analysis, or to sex-specific differences in the association.

Although our sample size was somewhat limited, we were able to replicate our results in an independent sample of women who later developed breast cancer, and to examine associations between the top SNPs and 25(OH)D concentrations in the same individuals at a later time point. All 25(OH)D measures were based on LC/MS, the current gold standard because of its improved precision (Farrell et al., 2012) and its ability to capture 25(OH)D_2_, 25(OH)D_3_, and epi-25(OH)D_3_, where the latter is an epimer of 25(OH)D_3_ that is thought to have nearly identical functionality (Cashman et al., 2014).

These results may not be generalizable to women of all races, particularly our findings for the *GC* gene and VDBP variants. Additionally, because our sample included women who had a sister with breast cancer, our effect estimates could be inflated for SNPs that interact with one or more breast cancer-related variants in their influence on vitamin D. However, we saw little difference in our results when we assessed women who later became cases vs. the random sub-cohort and our top hits and their effect estimates are consistent with findings of previous studies.

In this sample of women enrolled in the Sister Study, SNPs in *GC* and *CYP2R1* were strongly associated with serum 25(OH)D concentrations measured using LC/MS. Although these loci had been identified in earlier GWAS, these findings extend our understanding by pointing to possible roles for specific SNPs within these regions and further elucidating the importance of VDBP and cytochrome P450 enzymes in determining 25(OH)D concentrations. They may also help to identify individuals who are genetically predisposed to lower 25(OH)D and would most benefit from interventions to improve their circulating vitamin D levels.

## Author contributions

KO conceived and designed the research project, analyzed the data, wrote the paper, and has primary responsibility for final content. DS developed the overall research plan, oversaw data collection, and conducted study oversight. She also contributed to writing the manuscript. MS performed statistical analyses and contributed to the writing of the manuscript. QH helped with the interpretation of the results and the writing of the manuscript. JT helped to develop the overall research plan and write the paper. CW oversaw the overall research plan, the study design and statistical analysis, and helped to write the paper.

### Conflict of interest statement

The authors declare that the research was conducted in the absence of any commercial or financial relationships that could be construed as a potential conflict of interest.

## Footnotes

^1^https://www.ncbi.nlm.nih.gov/gap/phegeni

^2^Broad Institute The Genotype-Tissue Expression (GTEx) Project.

## Abbreviations

25(OH)D: 25-hydroxyvitamin D
AIMs: ancestry informative markers
CI: 95% confidence interval
GWAS: genome-wide association study
LC/MS: liquid chromatography-mass spectrometry
MAF: minor allele frequency
SNP: single nucleotide polymorphism
VDBP: vitamin D binding protein.
