# Supplementary material for: Genome-Wide Association Study of Serum 25-Hydroxyvitamin D in US Women
Source: Front Genet. 2018 Mar 1;9:67. doi: 10.3389/fgene.2018.00067 (PMC5838824; doi:10.3389/fgene.2018.00067)
Supplement: Supplementary file 1 [file DataSheet1.docx]

**Supplementary Table 1. Additional SNPs associated with serum 25(OH)D in the Sister Study (p>5x10^-8^ but q<0.10)**

| **SNP- rare allele** | **Chr:location^a^** | **Gene** | **Minor allele frequency, subcohort** | **HWE**  **p-value, subcohort** | **P-value, subcohort** | **P-value, cases** | **β^b^ (95% CI), pooled sample^c^** | **P-value, pooled sample^c^** | **q** | **P-value in follow-up study^c,d^** |
| --- | --- | --- | --- | --- | --- | --- | --- | --- | --- | --- |
| rs10766197-A | 11:14921880 |  | 0.44 | 0.10 | 1.4 x 10^-5^ | 0.001 | -1.3 (-1.8, -0.9) | 7.8 x 10^-8^ | 9.2 x 10^-4^ | 0.003 |
| rs4951247-A | 1:205597321 | *ELK4* | 0.21 | 0.04 | 2.6 x 10^-4^ | 4.6 x 10^-4^ | -1.5 (-2.1, -0.9) | 5.7 x 10^-7^ | 0.007 | 0.46 |
| rs78282269-A | 3:130029008 |  | 0.03 | 1.00 | 1.1 x 10^-4^ | 0.002 | 3.6 (2.1, 5.0) | 8.1 x 10^-7^ | 0.009 | 0.30 |
| rs10022720-G | 4:72743549 |  | 0.42 | 0.31 | 1.6 x 10^-4^ | 0.001 | 1.2 (0.7, 1.7) | 9.6 x 10^-7^ | 0.01 | 0.06 |
| rs4583526-A | 20:55270493 |  | 0.23 | 0.95 | 9.0 x 10^-4^ | 5.0 x 10^-4^ | 1.4 (0.8, 2.0) | 1.7 x 10^-6^ | 0.02 | 0.47 |
| rs9620654-A | 22:27430724 |  | 0.11 | 0.12 | 7.4 x 10^-5^ | 0.02 | -1.8 (-2.6, -1.1) | 3.0 x 10^-6^ | 0.03 | 5.4 x 10^-4^ |
| rs11023238-G | 11:14508698 | *COPB1* | 0.25 | 0.62 | 0.004 | 2.5 x 10^-4^ | -1.3 (-1.8, -0.7) | 4.3 x 10^-6^ | 0.04 | 0.15 |
| rs842990-C | 4:72578956 |  | 0.25 | 0.67 | 8.5 x 10^-4^ | 8.1 x 10^-4^ | 1.3 (0.7, 1.8) | 4.4 x 10^-6^ | 0.04 | 9.4 x 10^-4^ |
| rs16847200-A | 4:72751686 |  | 0.38 | 0.84 | 3.2 x 10^-4^ | 0.005 | 1.1 (0.6, 1.6) | 5.5 x 10^-6^ | 0.05 | 0.13 |
| rs6638987-C | X:9961481 |  | 0.10 | 0.51 | 0.003 | 6.3 x 10^-4^ | -1.9 (-2.7, -1.1) | 5.5 x 10^-6^ | 0.05 | 0.007 |
| rs360157-C | 11:9754221 | *MYO9B* | 0.42 | 0.29 | 0.001 | 0.002 | 1.1 (0.6, 1.6) | 7.2 x 10^-6^ | 0.06 | 9.4 x 10^-5^ |
| rs55634319-C | 19:44399097 |  | 0.12 | 0.17 | 0.002 | 0.002 | -1.7 (-2.4, -0.9) | 9.2 x 10^-6^ | 0.08 | 0.18 |
| rs12283049-G | 11:14690192 | *PDE3B* | 0.23 | 0.64 | 0.03 | 2.3 x 10^-5^ | -1.3 (-1.9, -0.7) | 9.4 x 10^-6^ | 0.08 | 0.001 |
| rs1451678-A | 11:14781621 | *PDE3B* | 0.49 | 0.67 | 0.002 | 0.001 | 1.1 (0.6, 1.6) | 9.7 x 10^-6^ | 0.08 | 0.07 |
| rs2276723-A | 3:192948186 |  | 0.32 | 0.45 | 9.8 x 10^-4^ | 0.006 | 1.1 (0.6, 1.6) | 1.1 x 10^-5^ | 0.09 | 0.004 |

All models adjusted for age at blood draw, self-reported race/ethnicity, and estimated ancestry proportions; MAF= minor allele frequency, HWE= Hardy-Weinberg equilibrium

^a^GRch37/hg19

^b^Change in 25(OH)D (in ng/mL) per copy of the minor allele

^c^Adjusting for case status

^d^Serum 25(OH)D levels measured in blood collected 3-10 years after baseline for the “Sisters Changing Lives” follow-up study

**Supplementary Table 2. Single nucleotide polymorphism associated with serum 25(OH)D levels at p<5 x 10^-8^ in the Sister Study (2003-2009)**

| **SNP-rare allele** | **Location** | **MAF,**  **sub-cohort** | **P-value,**  **sub-cohort** | **P-value, cases** | **β^a^ (95% CI), pooled sample^b^** | **P-value, pooled sample^b^** |
| --- | --- | --- | --- | --- | --- | --- |
| **Chromosome 4, *GC*** | |  |  |  |  |  |
| **rs4588-A** | **72618323** | **0.27** | **3.0 x 10^-23^** | **7.8 x 10^-19^** | **-3.5 (-4.0, -3.0)** | **1.9 x 10^-40^** |
| rs2282679-C | 72608383 | 0.26 | 6.2 x 10^-23^ | 6.3 x 10^-18^ | -3.4 (-3.9, -2.9) | 2.4 x 10^-39^ |
| rs1155563-G | 72643488 | 0.27 | 7.4 x 10^-20^ | 2.9 x 10^-12^ | -2.9 (-3.4, -2.4) | 3.6 x 10^-30^ |
| rs705120-A | 72614140 | 0.42 | 6.7 x 10^-12^ | 9.2 x 10^-12^ | -2.3 (-2.7, -1.8) | 1.8 x 10^-22^ |
| rs4694105-A | 72592214 | 0.31 | 8.6 x 10^-12^ | 1.1 x 10^-7^ | -2.2 (-2.7, -1.7) | 6.2 x 10^-18^ |
| rs2201124-A | 72597009 | 0.31 | 7.3 x 10^-12^ | 1.1 x 10^-7^ | -2.2 (-2.7, -1.7) | 7.1 x 10^-18^ |
| rs1526692-G | 72578724 | 0.40 | 3.6 x 10^-9^ | 1.0 x 10^-6^ | -1.8 (-2.3, -1.4) | 1.2 x 10^-14^ |
| rs6837549-A | 72596821 | 0.49 | 2.1 x 10^-6^ | 8.4 x 10^-6^ | 1.5 (1.1, 2.2) | 7.8 x 10^-11^ |
| rs13113067-A | 72739098 | 0.35 | 1.1 x 10^-7^ | 3.4 x 10^-4^ | -1.5 (-2.0, -1.1) | 2.3 x 10^-10^ |
| rs962227-A | 72707517 | 0.29 | 4.9 x 10^-7^ | 8.6 x 10^-4^ | -1.5 (-2.0, -1.0) | 2.5 x 10^-9^ |
| rs12639968-A | 72712872 | 0.18 | 9.6 x 10^-7^ | 4.0 x 10^-4^ | -1.8 (-2.3, -1.2) | 3.2 x 10^-9^ |
| rs10033936-G | 72743474 | 0.25 | 1.5 x 10^-6^ | 0.001 | -1.6 (-2.1, -1.0) | 1.2 x 10^-9^ |
| **Chromosome 11, *CYP2R1*** | |  |  |  |  |  |
| **rs117913124-A** | **14900931** | **0.02** | **8.1 x 10^-12^** | **0.002** | **-5.4 (-6.9, -3.9)** | **1.0 x 10^-12^** |
| rs12295888-G | 14450531 | 0.36 | 2.0 x 10^-5^ | 1.8 x 10^-9^ | -1.7 (-2.2, -1.2) | 3.4 x 10^-12^ |
| rs11023227-G | 14459087 | 0.36 | 1.6 x 10^-5^ | 2.4 x 10^-9^ | -1.7 (-2.2, -1.2) | 3.5 x 10^-12^ |
| rs10832275-C | 14478224 | 0.36 | 2.4 x 10^-5^ | 1.9 x 10^-9^ | -1.7 (-2.2, -1.2) | 3.6 x 10^-12^ |
| rs7121171-G | 14446420 | 0.36 | 1.9 x 10^-5^ | 2.9 x 10^-9^ | -1.7 (-2.2, -1.2) | 3.8 x 10^-12^ |
| rs11023223-G | 14457112 | 0.36 | 2.2 x 10^-5^ | 2.6 x 10^-9^ | -1.7 (-2.1, -1.2) | 5.1 x 10^-12^ |
| rs72261784-D | 14446060 | 0.36 | 2.9 x 10^-5^ | 2.1 x 10^-9^ | -1.7 (-2.1, -1.2) | 5.6 x 10^-12^ |
| rs10832268-A | 14465068 | 0.36 | 3.0 x 10^-5^ | 2.0 x 10^-9^ | -1.7 (-2.1, -1.2) | 5.7 x 10^-12^ |
| rs10832269-A | 14465069 | 0.36 | 3.1 x 10^-5^ | 2.4 x 10^-9^ | -1.7 (-2.1, -1.2) | 7.1 x 10^-12^ |
| rs11023246-G | 14536956 | 0.36 | 1.3 x 10^-4^ | 9.9 x 10^-10^ | -1.6 (-2.1, -1.2) | 2.0 x 10^-11^ |
| rs12794714-A | 14913575 | 0.42 | 2.5 x 10^-5^ | 4.2 x 10^-8^ | -1.6 (-2.1, -1.1) | 2.5 x 10^-11^ |
| rs201473898-A | 14893704 | 0.42 | 3.1 x 10^-5^ | 4.1 x 10^-8^ | -1.6 (-2.1, -1.1) | 3.5 x 10^-11^ |
| rs2305305-A | 14540942 | 0.36 | 2.0 x 10^-4^ | 1.2 x 10^-9^ | -1.6 (-2.1, -1.1) | 3.8 x 10^-11^ |
| rs10832294-A | 14747427 | 0.41 | 1.3 x 10^-4^ | 4.7 x 10^-8^ | -1.5 (-2.0, -1.1) | 2.5 x 10^-10^ |
| rs11023332-G | 14784110 | 0.42 | 2.9 x 10^-4^ | 2.2 x 10^-8^ | -1.5 (-2.0, -1.0) | 3.6 x 10^-10^ |
| rs1993116-A | 14910234 | 0.38 | 1.5 x 10^-5^ | 1.6 x 10^-5^ | 1.4 (1.0, 1.9) | 1.8 x 10^-9^ |
| rs10741657-A | 14914878 | 0.37 | 4.4 x 10^-5^ | 2.0 x 10^-5^ | 1.4 (0.9, 1.9) | 6.7 x 10^-9^ |
| rs11023203-A | 14409815 | 0.33 | 3.2 x 10^-4^ | 2.1 x 10^-6^ | -1.4 (-1.9, -0.9) | 1.3 x 10^-8^ |
| rs10766188-G | 14660826 | 0.34 | 0.002 | 3.4 x 10^-7^ | -1.4 (-1.9, -0.9) | 1.7 x 10^-8^ |

All models adjusted for age at blood draw, self-reported race/ethnicity, estimated ancestry proportions, total estimated vitamin D intake (dietary plus supplement), and time spent outdoors (hours/year)

^a^Change in 25(OH)D (in ng/mL) per copy of the minor allele

^b^Adjusting for case status

**Supplementary Figure 1a. Linkage disequilibrium map showing correlations (R^2^) for SNPs on chromosome 4 near rs4588 (*GC*) among participants in the sub-cohort**

**
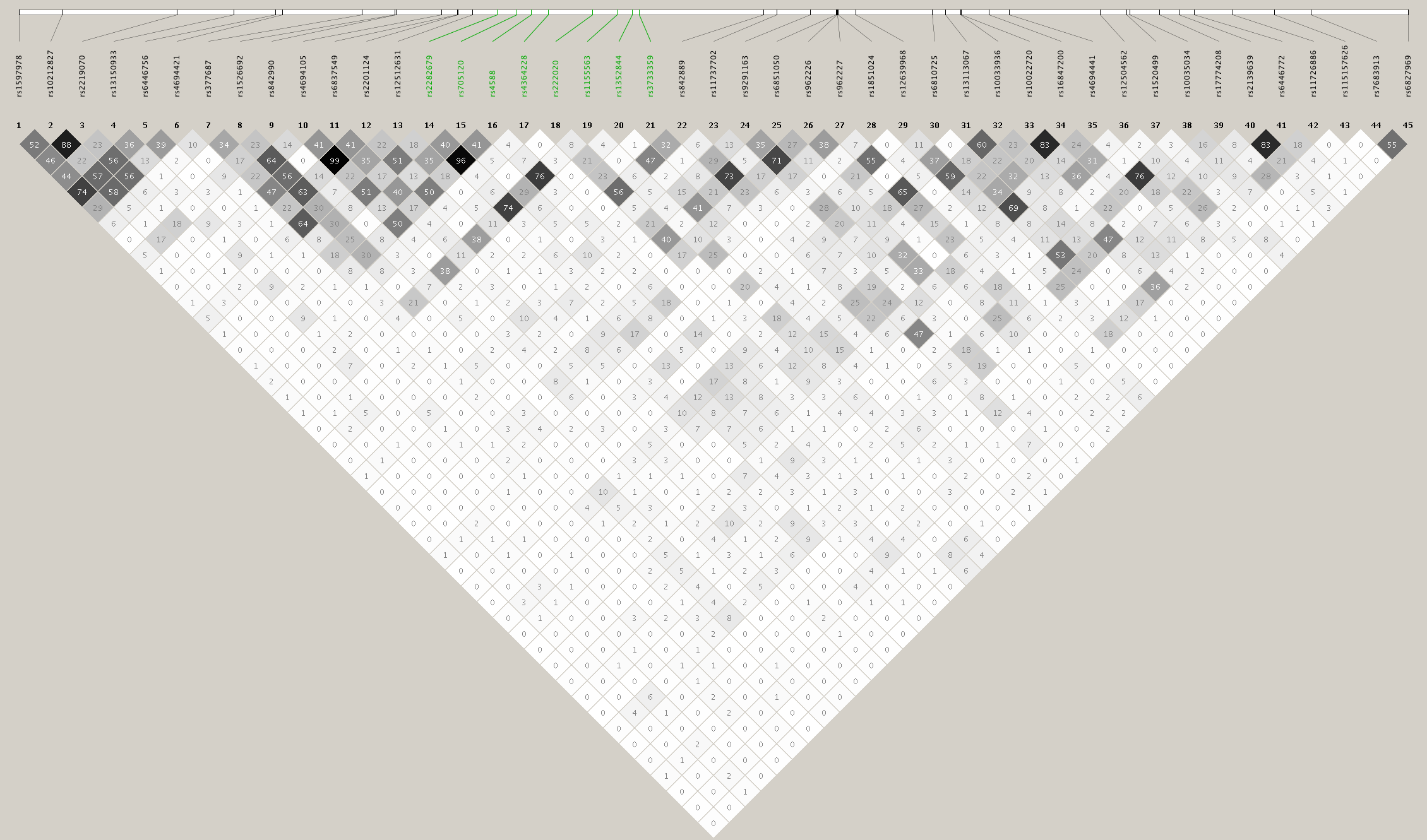
**

SNPs highlighted in green are located in *GC*.

**Supplementary Figure 1b. Linkage disequilibrium map showing correlations (R^2^) for SNPs on chromosome 4 near rs4588 (*GC*) among participants in the sub-cohort; SNPs with statistically significant (p<5x10^-8^) associations with 25(OH)D**

**
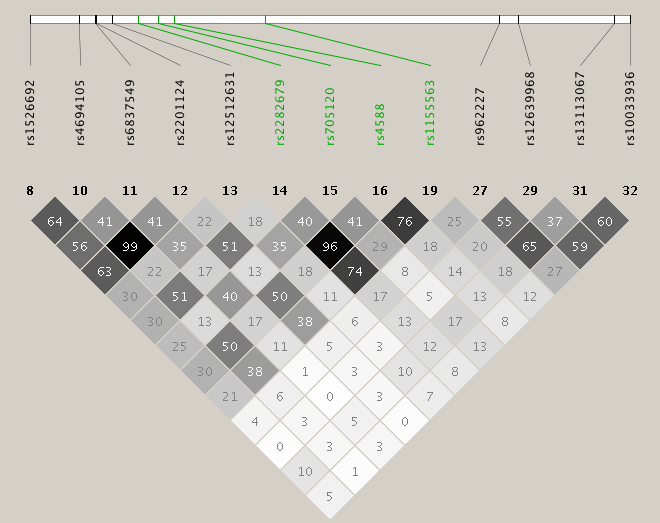
**

SNPs highlighted in green are located in *GC*.

**Supplementary Figure 1c. Linkage disequilibrium map showing correlations (R^2^) for SNPs on chromosome 4 near rs4588 (*GC*) among participants in the sub-cohort; SNPs used for haplotype analysis (p<1x10^-10^) and not in high linkage disequilibrium with a more informative SNP (r^2^<0.80))**

**
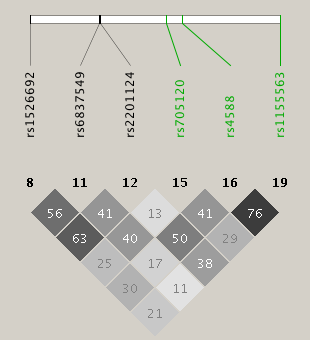
**

SNPs highlighted in green are located in *GC*.

**Supplementary Figure 2a. Linkage disequilibrium map showing correlations (R^2^) for SNPs on chromosome 11 near rs12794714 (*CYP2R1*) among participants in the sub-cohort**

**
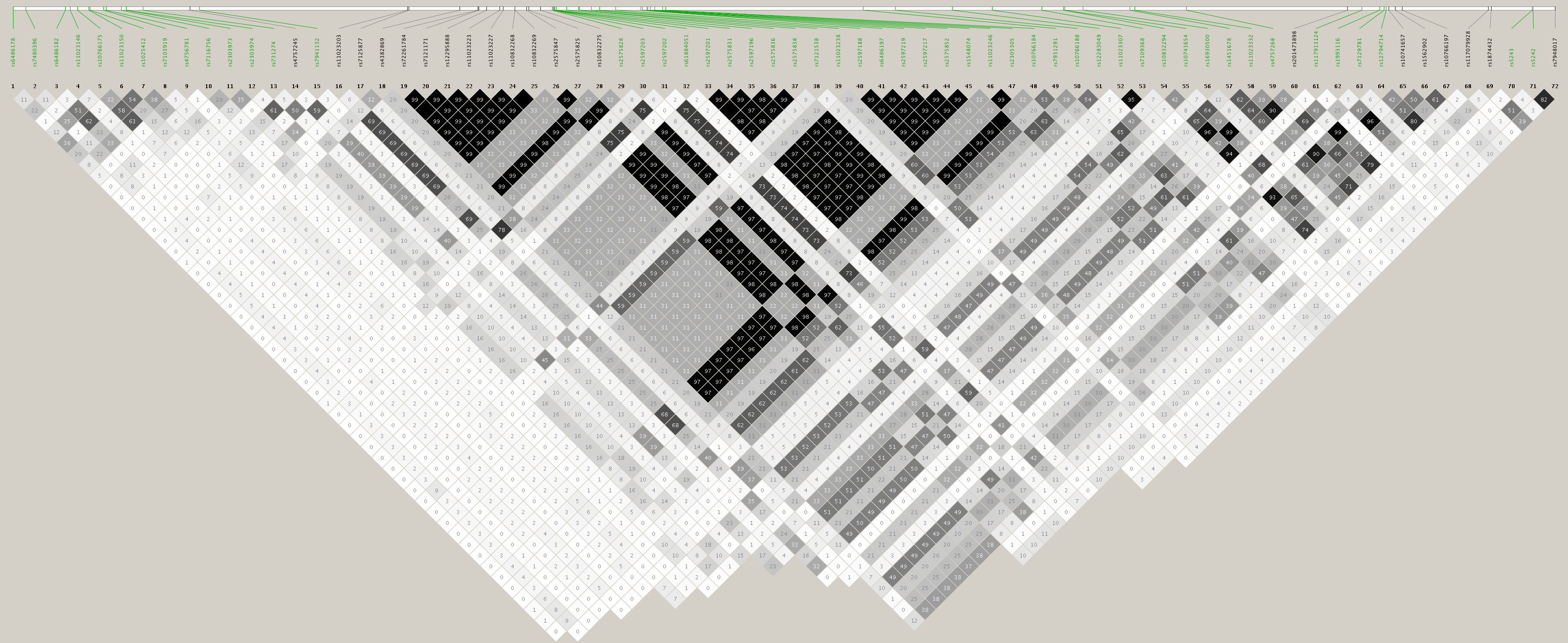
**

SNPs shown in green are located in genes. Correlations not shown for SNPs located >500Kb apart.

**Supplementary Figure 2b. Linkage disequilibrium map showing correlations (R^2^) for SNPs on chromosome 11 near rs12794714 (*CYP2R1*) among participants in the sub-cohort; genome-wide significant SNPs (p<5x10^-8^)**

**
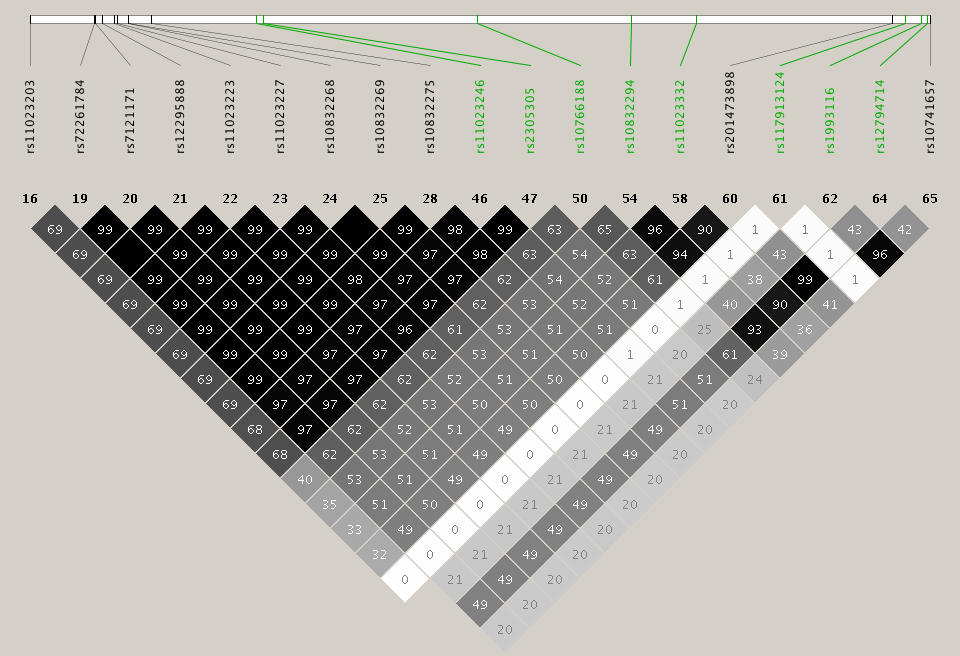
**

SNPs shown in green are located in genes (*PDE3B*: rs11023235, rs2305305, rs10766188, and rs11023332; *CYP2R1*: rs117913124, rs1993116, and rs12794714). Correlations not shown for SNPs located >500Kb apart.

**Supplementary Figure 2c. Linkage disequilibrium map showing correlations (R^2^) for SNPs on chromosome 11 near rs12794714 (*CYP2R1*) among participants in the sub-cohort; SNPs included in haplotype analysis (MAF >5%; p<5x10^-8^), not in high linkage disequilibrium with a more informative SNP (R^2^<0.80)).**


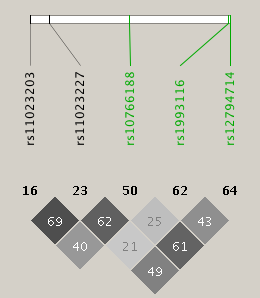


SNPs shown in green are located in genes (*PDE3B*: rs10766188; *CYP2R1*: rs1993116 and rs12794714). Correlations not shown for SNPs located >500Kb apart (R^2^ for rs11023203 and rs1993116=0.14; R^2^ for rs11023203 and rs12794714=0.33).

**Supplementary Table 3. Minor allele frequencies by race/ethnicity in sub-cohort**

| **SNP** | **Overall MAF** | **MAF, non-Hispanic whites** | **MAF, African-Americans** | **MAF, Hispanic** | **MAF, Other** |
| --- | --- | --- | --- | --- | --- |
| rs4588 (*GC*) | 0.27 | 0.28 | 0.11 | 0.20 | 0.22 |
| rs705120 (*GC*) | 0.42 | 0.42 | 0.44 | 0.36 | 0.43 |
| rs12794714 (*CYP2R1*) | 0.42 | 0.44 | 0.17 | 0.42 | 0.39 |
| rs11023227 (*CYP2R1*) | 0.36 | 0.38 | 0.16 | 0.38 | 0.41 |
| rs117913124 (*CYP2R1*) | 0.02 | 0.03 | 0.01 | 0.00 | 0.02 |
